# Macrophage morphology in the tumor microenvironment predicts metachronous liver metastasis in gastric cancer: establishment and validation of a predictive model

**DOI:** 10.3389/fimmu.2026.1770436

**Published:** 2026-06-05

**Authors:** Siyuan Wang, Gaozan Zheng, Xinyu Qiao, Ye Tian, Ruikai Li, Hanjun Dan, Yumao Yang, Lili Duan, Liaoran Niu, Jianting Yang, Fengsu Wu, Jianyong Zheng, Fan Feng

**Affiliations:** 1 Department of Digestive Surgery, Xijing Hospital of Digestive Diseases, Fourth Military Medical University, Shaanxi, China; 2 Department of General Surgery, The 989th Hospital of the Joint Logistics Support Force of People‘s Liberation Army (PLA), Henan, China; 3 Department of Gastroenterology, Pingdingshan Medical District, 989 Hospital of People‘s Liberation Army (PLA) Joint Logistics Support Force, Henan, China

**Keywords:** gastric cancer, macrophage morphology, metachronous liver metastasis, nomogram, tumor microenvironment

## Abstract

**Background:**

Gastric cancer patients with metachronous liver metastasis (MLM) generally pose a significant clinical challenge, with 5-year survival below 10%. Macrophages are key components of a tumoral microenvironment, but the predictive value of their morphological features for MLM remains unexplored. This study aimed to construct a risk predictive model for MLM based on macrophage morphology in the tumor microenvironment.

**Methods:**

To reduce major baseline imbalances between patients with and without MLM, propensity score matching (PSM) was performed. After matching, a retrospective analysis of 233 gastric cancer patients who underwent radical surgery between 2016 and 2020 was conducted. Macrophage morphological parameters in different tissues were quantified by Qupath. Patients were randomly divided into training (70%) and validation (30%) datasets. The optimal cutoff values for the continuous variables were determined using the Youden index. Univariate and multivariate logistic regression analyses in the training set were used to identify risk factors for MLM. A nomogram was constructed for clinical applications. The model’s value was validated through receiver operating characteristic (ROC) curves, calibration curves, and decision curve analysis (DCA).

**Results:**

The incidence of MLM was similar in the training (38.7%, 63/163) and validation cohorts (32.9%, 23/70). The optimal cut off value of macrophage areas and perimeters in the tumor invasive front (IF) region, tumor region, peritumoral stroma (PS) region were 76.75 μm^2^, 35.77 μm, 71.35 μm^2^, 32.00 μm, 74.13 μm^2^, 31.32 μm. Multivariate analysis revealed that only the area and perimeter of macrophages in the IF region, the area of macrophages in the tumor region were independent predictive factors for MLM. These factors were incorporated into a predictive nomogram. The model demonstrated good discrimination, with an area under the curve (AUC) of 0.835 in the training set and 0.798 in the validation set. Calibration and decision curve analyses confirmed its clinical utility.

**Conclusion:**

Macrophage morphology parameters, particularly in the IF and tumor regions, are significantly associated with MLM in gastric cancer. The morphology-based nomogram developed in this study provides an exploratory tool for risk stratification and may help inform individualized postoperative surveillance.

## Introduction

Gastric cancer remains a major global health burden, ranking as the fifth most frequently diagnosed malignancy and the fifth leading cause of cancer-related mortality worldwide (1). Despite advances in surgery, chemotherapy, and targeted therapies, patients with gastric cancer still experience poor survival outcomes and high mortality rates (2). One primary contributor to this adverse outcome is the tendency of gastric cancer to metastasize distantly with common sites include liver, peritoneum, and bone (3). The liver is one of the most common sites of distant metastasis, with an incidence ranging from 5% to 37%, and patients with liver metastasis exhibit extremely poor prognosis, with five-year survival rates below 10% (4–8). Liver metastasis after gastrectomy is generally categorized according to its timing as either synchronous liver metastasis (SLM) or metachronous liver metastasis (MLM), with the latter defined as liver metastasis diagnosed more than six months after surgery (9). Although numerous studies (10–12) have investigated the prognosis of gastric cancer and the prediction of SLM, few have focused on predicting MLM. Our previous research indicated that patients with elevated preoperative peripheral blood monocytes and reduced lymphocyte counts were at an increased risk of developing MLM (13). Since peripheral blood monocytes are the major precursor cells of macrophages in the tumor microenvironment and can reflect systemic immune alterations (14, 15), it is reasonable to hypothesize that the phenotypic and functional characteristics of macrophages in the gastric cancer microenvironment may also be closely related to the occurrence of MLM.

Macrophages, essential elements of the tumor microenvironment, pave the way to tissue invasion and intravasation and provide a nurturing microenvironment for tumor metastasis, serving as a component of the cancer cell niche at distant sites (16). Macrophages exhibit pronounced heterogeneity (17), they exhibit remarkable plasticity and diversity in function, which is often reflected in their morphological characteristics (18). Costa et al. (19) assessed macrophages in colorectal liver metastases (CLM) and correlated their morphometry with clinical prognosis, showing that larger TAMs (L-TAMs) predict poorer survival. However, the specific contribution of macrophage morphology to predicting MLM in gastric cancer remains unexplored. Prior studies (20–22) were largely focused on macrophage density or conventional polarization markers in primary tumors, while morphological features such as cell area and perimeter have received little attention as quantifiable indicators of macrophage functional diversity. Beyond CD68, given that prior study has suggested CD163 is more uniformly distributed on the cell surface (16), we included CD163 staining to explore whether morphology linked to a specific polarization phenotype would provide superior predictive value for MLM.

Based on this, our study aimed to investigate the morphological features of macrophages in primary gastric cancer tissues, with a focus on different histological regions, such as the IF region, tumor region and PS region, to evaluate their predictive value for MLM. We hypothesized that analyzing macrophage morphotypes in primary gastric tumors could provide a novel, accessible tool for MLM risk assessment.

## Materials and methods

### Patients

**Figure 1:**
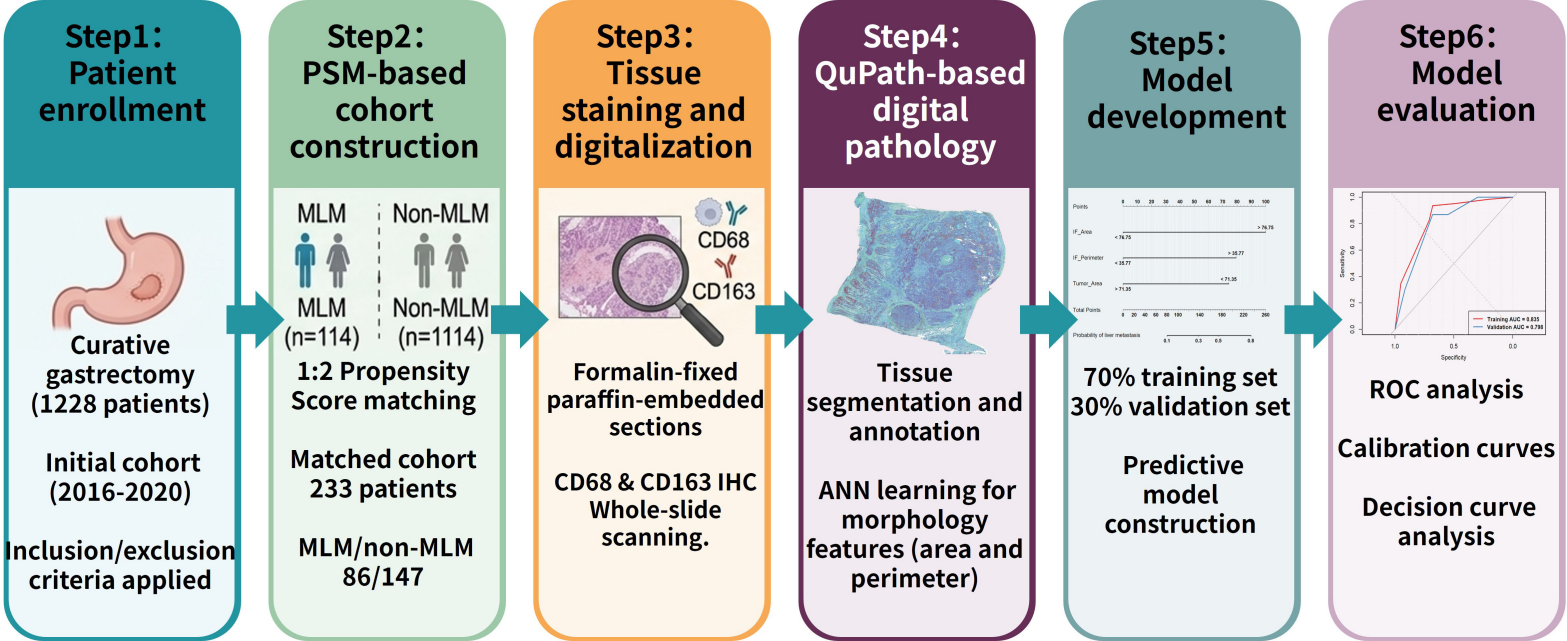
The study design is presented in six sequential steps. (1) Patient enrollment: a total of 1228 patients with gastric cancer who underwent curative gastrectomy between 2016 and 2020 were screened according to predefined inclusion and exclusion criteria. (2) PSM-based cohort construction: to reduce baseline imbalance between MLM and non-MLM groups, 1:2 propensity score matching (PSM) was performed, resulting in a matched cohort of 233 patients (86 MLM and 147 non-MLM cases). (3) Tissue staining and digitalization: formalin-fixed paraffin-embedded sections were stained with CD68 and CD163 antibodies, followed by whole-slide scanning. (4) QuPath-based digital pathology: tissue segmentation and annotation were performed in QuPath, and artificial neural network (ANN) learning was applied to extract macrophage morphological features (area and perimeter) in different histological regions. (5) Model development: the matched cohort was randomly divided into a 70% training set and a 30% validation set, and a predictive nomogram was constructed based on macrophage morphological parameters. (6) Model evaluation: the performance of the nomogram was assessed using receiver operating characteristic (ROC) analysis, calibration curves, and decision curve analysis (DCA). MLM, metachronous liver metastasis; IHC, immunohistochemistry.

This retrospective study enrolled patients treated at the Department of Digestive Surgery, Xijing Hospital of Digestive Diseases, between December 2016 to December 2020. The inclusion criteria were as follows: (1) histologically verified gastric adenocarcinoma; (2) received curative gastrectomy with R0 resection; (3) availability of complete clinical data; and (4) well-preserved postoperative pathological tissue sections. The exclusion criteria were as follows: (1) liver metastasis occurring within 6 months after surgery; (2) prior or synchronous other malignancies; (3) received neoadjuvant therapy prior to surgery; (4) subsequent non-liver metastatic progression; (5) no postoperative liver metastasis with follow-up <36 months. The study was conducted in accordance with the Declaration of Helsinki and received ethical approval (KY20252237-C-1) from the Ethics Committee of the Xijing Hospital. The requirement for informed consent was waived due to the retrospective nature of the study. The analysis workflow is shown in the flowchart in **Figure 1**. To reduce major baseline imbalances between patients with and without MLM and to allow a more focused exploration of the association between macrophage morphological characteristics in the primary tumor microenvironment and subsequent MLM, we performed propensity score matching (PSM).

### Clinical information and follow-up

Clinical variables, including gender, age, tumor location, tumor size, pathological type, surgical method, T stage, N stage, TNM stage, neural invasion, lymphatic vascular invasion and surgical method were retrospectively extracted from medical records. Patients were followed every 3 months for the first 3 years and every 6 months thereafter. Liver metastasis was diagnosed by contrast-enhanced CT. Non-MLM patients were followed for at least 3 years post-surgery. Follow-up time was calculated from the day after surgery until either MLM diagnosis or study endpoint.

### Immunohistochemistry

To characterize the macrophage morphology in different regions, paraffin-embedded cancer specimens were serially sectioned at a thickness of 3 μm. After deparaffinization in xylene and rehydration through a graded alcohol series, endogenous peroxidase activity was quenched by incubating the slides with 3% hydrogen peroxide. To suppress endogenous peroxidase activity, the slides were treated with 10% goat plasma. Subsequently, the slides were incubated overnight at 4 °C with primary antibodies targeting human CD68 (1:500, Servicebio, China) and CD163 (1:500, Servicebio, China). Following primary antibody incubation, the sections were successively exposed to a biotinylated secondary antibody and streptavidin–horseradish peroxidase complex. Antigen–antibody interactions were visualized using diaminobenzidine (DAB) as the chromogen, complemented by hematoxylin counterstaining to highlight the cell nuclei. The slides were digitized using a Pannoramic Digital Slide Scanner from 3DHISTECH, Sysmex, Hungary.

### Image analysis

QuPath (version 0.6.0.) software (23) was used to obtain accurate measurements of positive macrophage parameters in different tissue regions (**Supplementary Figure 1**). The tissue classifier was developed and internally applied within a standardized institutional workflow. First, standardized color deconvolution was performed with fixed stain vectors for hematoxylin and DAB, alongside consistent optical density thresholds. Morphological filters and manual annotation were used to exclude debris, necrotic tissue, background noise, and artifacts. For tissue segmentation, SLIC superpixel segmentation was applied with Gaussian sigma = 5 μm, superpixel spacing = 50 μm, number of iterations = 10, and regularization = 0.25 using color−deconvolved channels. Inspired by the study conducted by Zhao et al (24), Gastric cancer tissues were grouped into seven classifications: tumor (TUM), invasive front (IF), peritumoral stroma (PS), mucin (MUC), smooth muscle (MUS), normal mucosa (NOR), and background including blank areas, debris and necrotic tissue (BAC). TUM was defined as areas predominantly composed of malignant epithelial cells. The IF region was defined as the area within 500 μm of the tumor boundary, according to the study by Wu et al. (25). PS was defined as non-neoplastic stromal tissue adjacent to tumor nests and enriched in fibrovascular and inflammatory components. MUC comprised mucin pools, MUS represented smooth muscle bundles, NOR represented histologically preserved non-neoplastic gastric mucosa, and BAC included blank areas, debris, necrosis, and obvious artifacts.

Specifically, a learning region with a total area of 0.685 mm² was identified under a 10× magnification field in QuPath for tissue annotation. For each slide, typical regions representing the different tissue categories were manually delineated, ensuring that each category was annotated at least five times per slide (**Supplementary Figure 2**). These annotated regions were then incorporated into the artificial neural network (ANN) training model for learning. After the classifier model was trained, its performance was validated on the whole slide from which the annotations were derived, the classifier was subsequently applied to other slides. All annotations were performed by two pathologists with over 10 years of diagnostic experience using a double-blind approach, any discrepancies in annotations were resolved by another senior pathologist for final confirmation. They were blinded to all patient information and clinical outcomes associated with the immunohistochemical sections.

At the cellular level, the unified detection parameters across all CD68 and CD163 slides were as follows: requested pixel size = 0.5 μm, background radius = 8 μm, sigma = 1.5 μm, minimum area = 10 μm², maximum area = 400 μm², intensity threshold = 0.02, maximum background intensity = 2, cell expansion radius = 2 μm, a single positivity threshold of 0.2. Then, positive macrophages across the entire slide were first identified using the positive cell detection module. The trained tissue classifier was subsequently transferred to the cellular level to assign each positive macrophage to a corresponding tissue region. Finally, the output data were extracted for downstream analysis. Representative whole−slide immunohistochemical images, tissue region segmentation maps, and annotated CD68^+^ and CD163^+^ macrophages distribution are provided in **Supplementary Figure 3** to illustrate the quality of staining and the performance of our automated digital pathology analytical pipeline.

### Propensity score matching analysis

**Figure 2:**
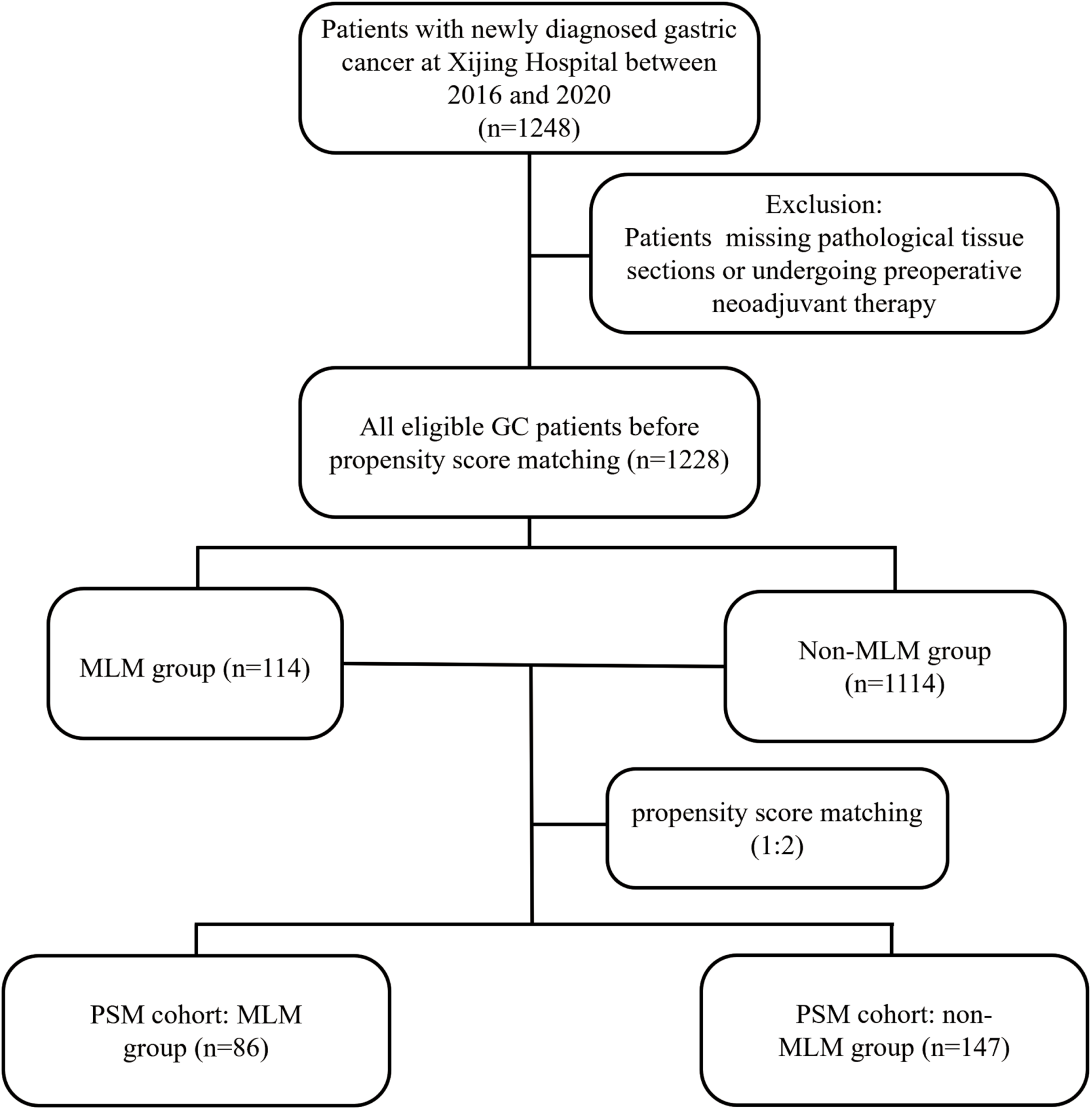
Flowchart of patient inclusion, exclusion, and cohort construction.

To reduce major baseline imbalances between patients with and without MLM and to allow a more focused exploration of the association between macrophage morphological characteristics in the primary tumor microenvironment and subsequent MLM, we performed PSM. A 1:2 matching ratio with a caliper width of 0.02 was applied, with the specific matching parameters as follows: age, tumor size, gender, tumor location, pathological type, T stage, N stage, TNM stage, neural invasion, lymphovascular invasion, and surgical method. The matching process and cohort selection are illustrated in **Figure 2**.

### Statistical analysis

Statistical analysis was performed using R (version 4.4.1) and SPSS (version 27.0). Continuous variables were converted to categorical variables to facilitate the interpretation of results, and optimal cut-off values for macrophage parameters were determined using ROC curves with the maximal Youden index (26). ROC analyses for individual morphometric variables were performed in the training cohort. The optimal cut-off value for each variable was determined by maximizing the Youden index (sensitivity + specificity – 1). The derived cut-offs were subsequently applied to both the training and validation cohorts. Categorical variables were expressed as frequencies and percentages, with chi-square tests used. In the training dataset, univariate analysis was initially used to evaluate risk factors for MLM. Variables with p<0.1 in univariate analysis were included in the multivariate logistic regression model using forward stepwise selection. A prediction nomogram was constructed using the results of the multivariate regression analysis, and its predictive accuracy was assessed by discrimination (AUC) and calibration (bootstrapping with 1000 resamples). Calibration plots were used to compare observed and predicted probabilities, and clinical utility was assessed through DCA curves. Statistical significance was set at p<0.05 for two-tailed tests.

## Results

### Baseline characteristics before and after propensity score matching

A total of 1228 patients including 114 MLM patients and 1114 non-MLM patients were included in our study. As shown in **Supplementary Table 1**, there were significant differences in tumor size, gender, tumor location, pathological type, T stage, N stage, TNM stage, neural invasion, lymphovascular invasion, surgical method between the MLM and non-MLM groups (all P < 0.05). PSM was implemented to balance baseline characteristics between the two group. After matching, 86 MLM patients and 147 non-MLM patients were included. Baseline characteristics were well-balanced between groups (all P > 0.05, **Table 1**).

**Table 1:** Baseline clinical characteristics between MLM and non-MLM group after PSM.

Characteristics	Non-MLM(n=147) (%)	MLM(n=86) (%)	P	SMD
Age (year (SD))	58.27 ± 10.66	58.74 ± 10.10	0.740	0.045
Tumor size (cm (SD))	5.04 ± 2.33	4.97 ± 1.99	0.812	0.033
Gender			0.863	0.051
Male	129 (87.8)	74 (86.0)		
Female	18 (12.2)	12 (14.0)		
Tumor location			0.716	0.149
Upper third	49 (33.3)	30 (34.9)		
Middle third	30 (20.4)	18 (20.9)		
Lower third	67 (45.6)	36 (41.9)		
Entire	1 (0.7)	2 (2.3)		
Pathological type			0.163	0.265
Moderately differentiated	39 (26.5)	14 (16.3)		
Poorly differentiated	91 (61.9)	63 (73.3)		
Signet ring cell/Mucinous /undifferentiated	17 (11.6)	9 (10.5)		
T stage			0.537	0.205
T1	2 (1.4)	2 (2.3)		
T2	12 (8.2)	8 (9.3)		
T3	67 (45.6)	31 (36.0)		
T4	66 (44.9)	45 (52.4)		
N stage			0.652	0.177
N0	29 (19.7)	16 (18.6)		
N1	31 (21.1)	14 (16.3)		
N2	40 (27.2)	22 (25.6)		
N3	47 (32.0)	34 (39.5)		
TNM stage			0.585	0.142
I	5 (3.4)	3 (3.5)		
II	47 (32.0)	22 (25.6)		
III	95 (64.6)	61 (70.9)		
Neural invasion			0.860	0.055
Negative	13 (8.8)	9 (10.5)		
Positive	134 (91.2)	77 (89.5)		
Lymphovascular invasion			0.627	0.088
Negative	38 (25.9)	19 (22.1)		
Positive	109 (74.1)	67 (77.9)		
Surgical method			0.746	0.104
Proximal Gastrectomy	14 (9.5)	7 (8.1)		
Distal Gastrectomy	67 (45.6)	36 (41.9)		
Total Gastrectomy	66 (44.9)	43 (50.0)		

### Morphological characterization and feature extraction of macrophages

To validate the consistency between manual and automated detection, random regions of interest (ROI) with a total area of 0.17125 mm² were selected under 20× magnification for morphological outlining of macrophages in QuPath. Fifty patients were randomly chosen, and the same ROI regions were analyzed both manually and using automated macrophage outlining. Excellent agreement was observed between the two methods, with Pearson’s correlation coefficients of 0.904 for macrophage area and 0.921 for perimeter (both p < 0.001) (**Supplementary Figure 4**).

Following validation, QuPath was applied to automatically outline positive macrophages across all tissue regions in 233 patients. For each patient, six morphological features — the area and perimeter of macrophages in the IF, tumor, and PS regions — were collected from all annotated regions. For each annotated region, morphological features of all positive macrophages were collected, and the mean value per region was calculated for subsequent analyses.

### Study population and determination of cut-off values

The clinicopathological characteristics were summarized in **Table 2**. A total of 203 males and 30 females were included in the cohort, with a mean age of 58 years. The median follow-up duration was 63 months (range 8-88). Patients were randomly allocated to a training set (n=163) and a validation set (n=70) in a ratio of 7:3. The incidence of MLM was comparable between the training (38.7%, 63/163) and validation set (32.9%, 23/70). The optimal cut-off values for age, tumor size, were 55years, 3.75cm.

**Table 2:** Characteristics of the patients in the training and validation cohorts.

Characteristics	Overall(n=233)(%)	Training(n=163)(%)	Validation(n=70)(%)	X^2^	P
Age (years)				1.365	0.243
<55	76 (32.6)	57 (35.0)	19 (27.1)		
≥55	157 (67.4)	106 (65.0)	51 (72.9)		
Size (cm)				0.537	0.464
<3.75	72 (30.9)	48 (29.4)	24 (34.3)		
≥3.75	161 (69.1)	115 (70.6)	46 (65.7)		
Gender				0.187	0.666
Male	203 (87.1)	141 (86.5)	62 (88.6)		
Female	30 (12.9)	22 (13.5)	8 (11.4)		
Tumor location				3.231	0.357
Upper third	79 (33.9)	57 (35.0)	22 (31.4)		
Middle third	48 (20.6)	36 (22.1)	12 (17.1)		
Lower third	103 (44.2)	67 (41.1)	36 (51.4)		
Entire	3 (1.3)	3 (1.84)	0 (0.00)		
Pathological type				0.337	0.845
Moderately differentiated	53 (22.7)	38 (23.3)	15 (21.4)		
Poorly differentiated	154 (66.1)	108 (66.3)	46 (65.7)		
Signet ring cell/Mucinous /undifferentiated	26 (11.2)	17 (10.4)	9 (12.9)		
Surgical Method				1.406	0.495
Proximal Gastrectomy	21 (9.0)	17 (10.4)	4 (5.8)		
Distal Gastrectomy	103 (44.2)	70 (43.0)	33 (47.1)		
Total Gastrectomy	109 (46.8)	76 (46.6)	33 (47.1)		
T stage				2.633	0.452
T1	4 (1.7)	4 (2.5)	0 (0.0)		
T2	20 (8.6)	15 (9.2)	5 (7.1)		
T3	98 (42.1)	70 (42.9)	28 (40.0)		
T4	111 (47.6)	74 (45.4)	37 (52.9)		
N stage				2.903	0.407
N0	45 (19.3)	33 (20.2)	12 (17.1)		
N1	45 (19.3)	33 (20.2)	12 (17.1)		
N2	62 (26.6)	46 (28.2)	16 (22.9)		
N3	81 (34.8)	51 (31.3)	30 (42.9)		
TNM stage				0.442	0.802
I	8 (3.4)	6 (3.7)	2 (2.9)		
II	69 (29.6)	50 (30.7)	19 (27.1)		
III	156 (67.0)	107 (65.6)	49 (70.0)		
Neural invasion				3.111	0.078
Negative	22 (9.4)	19 (11.7)	3 (4.3)		
Positive	211 (90.6)	144 (88.3)	67 (95.7)		
Lymphovascular invasion				0.389	0.533
Negative	57 (24.5)	38 (23.3)	19 (27.1)		
Positive	176 (75.5)	125 (76.7)	51 (72.9)		
Liver metastases				0.706	0.401
No	147 (63.1)	100 (61.3)	47 (67.1)		
Yes	86 (36.9)	63 (38.7)	23 (32.9)		
CD68 IF Area (μm^2^)				1.960	0.162
<76.75	81 (34.8)	52 (31.9)	29 (41.4)		
≥76.75	152 (65.2)	111 (68.1)	41 (58.6)		
CD68 IF Perimeter (μm)				0.002	0.966
<35.77	117 (50.2)	82 (50.3)	35 (50.0)		
≥35.77	116 (49.8)	81 (49.7)	35 (50.0)		
CD68 Tumor Area (μm^2^)				0.025	0.875
<71.35	95 (40.8)	67 (41.1)	28 (40.0)		
≥71.35	138 (59.2)	96 (58.9)	42 (60.0)		
CD68 Tumor Perimeter (μm)				0.010	0.921
<32.00	91 (39.1)	64 (39.3)	27 (38.6)		
≥32.00	142 (60.9)	99 (60.7)	43 (61.4)		
CD68 PS Area (μm^2^)				0.003	0.953
<74.13	216 (92.7)	151 (92.6)	65 (92.9)		
≥74.13	17 (7.3)	12 (7.4)	5 (7.1)		
CD68 PS Perimeter (μm)				0.430	0.512
<31.32	53 (22.7)	39 (23.9)	14 (20.0)		
≥31.32	180 (77.3)	124 (76.1)	56 (80.0)		
CD163 IF Area (μm^2^)				3.127	0.077
<80.12	93 (39.9)	59 (36.2)	34 (48.6)		
≥80.12	140 (60.1)	104 (63.8)	36 (51.4)		
CD163 IF Perimeter (μm)				3.438	0.064
<36.96	130 (55.8)	84 (51.5)	46 (65.7)		
≥36.96	103 (44.2)	79 (48.5)	24 (34.3)		
CD163 Tumor Area (μm^2^)				0.358	0.550
<77.92	103 (61.4)	98 (60.1)	45 (64.3)		
≥77.92	90 (38.6)	65 (39.9)	25 (35.7)		
CD163 Tumor Perimeter (μm)				1.922	0.166
<32.20	75 (32.2)	57 (35.0)	18 (25.7)		
≥32.20	158 (67.8)	106 (65.0)	52 (74.3)		
CD163 PS Area (μm^2^)				0.797	0.372
<70.50	140 (60.1)	101 (62.0)	39 (55.7)		
≥70.50	93 (39.9)	62 (38.0)	31 (44.3)		
CD163 PS Perimeter (μm)				1.922	0.166
<33.47	139 (60.7)	102 (62.6)	37 (52.9)		
≥33.47	94 (40.3)	61 (37.4)	33 (47.1)		

The optimal cut-off values for the areas and perimeters of CD68^+^ macrophages group in the IF, TUM, PS region were 76.75 μm^2^, 35.77 μm, 71.35 μm^2^, 32.00 μm, 74.13 μm^2^, 31.32 μm, respectively. The morphological features of CD163^+^ macrophages were also evaluated. The optimal cut-off values for the areas and perimeters of CD163^+^ macrophages group in the IF, TUM, PS region were 80.12 μm², 36.96 μm, 77.92 μm², 32.20 μm, 70.50 μm², 33.47 μm, respectively. The optimal cut-off values, corresponding AUCs, sensitivities, specificities, and Youden indices for the evaluated morphometric parameters are summarized in **Supplementary Table 2**, and the corresponding ROC curves are shown in **Supplementary Figure 5**.

### Establishment of the prediction nomogram for MLM

In the CD163^+^ macrophage analysis, only IF area remained independently associated with MLM in multivariable analysis. Complete univariate results for CD163^+^ parameters are summarized in **Supplementary Table 3**. Therefore, CD163^+^ parameters were not included in the final nomogram due to the lack of multiple independent predictors. For exploratory comparison, we also constructed a CD163-based model, which included only one retained variable and showed lower predictive performance than the CD68-based nomogram; these results are provided in the **Supplementary Figure 6**. The following analyses and the resulting prediction model are based on CD68^+^ macrophage morphological parameters, whereas the CD163-based analysis was retained only as a supplementary exploratory comparison.

**Figure 3:**
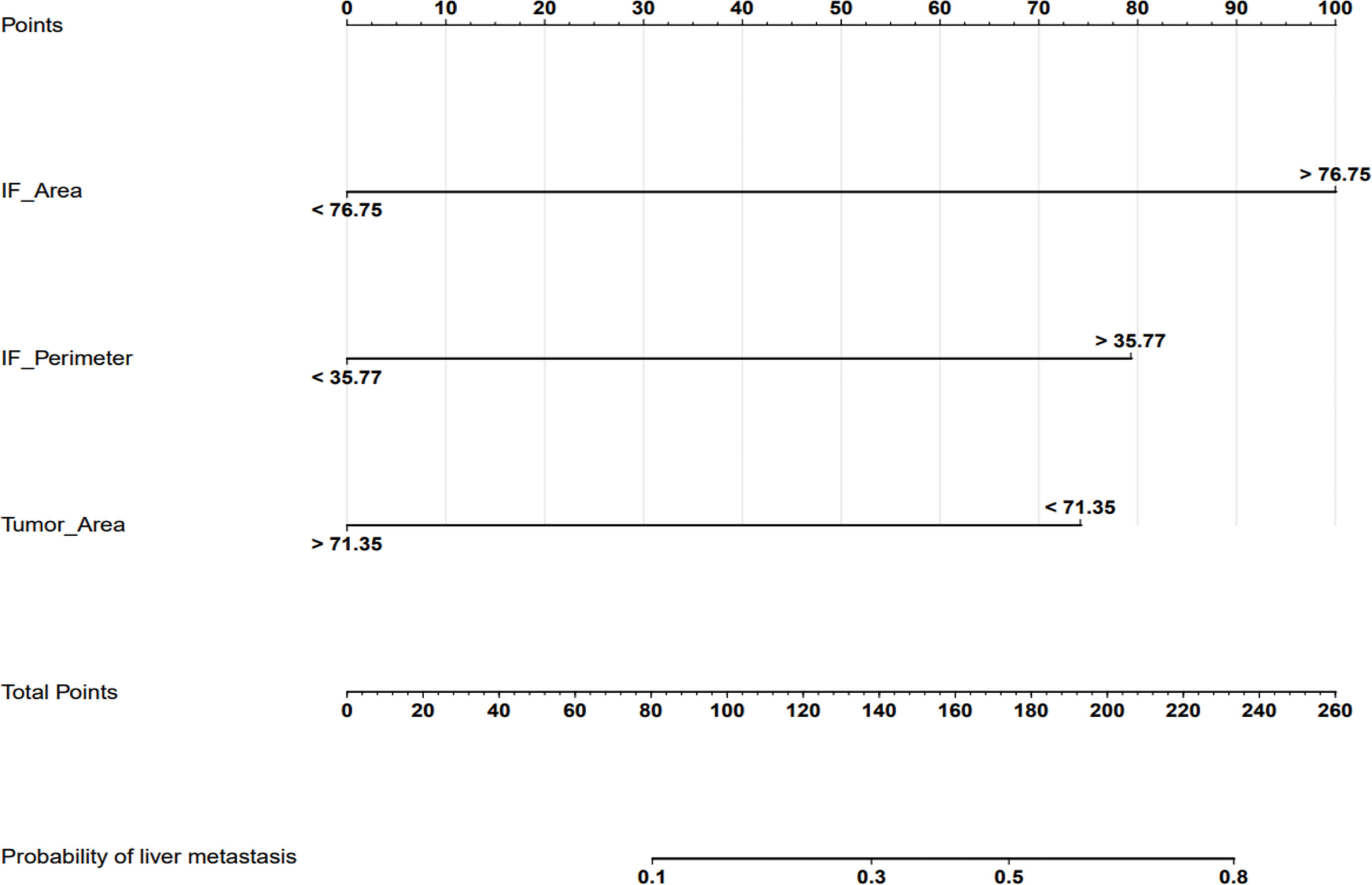
Nomogram for predicting metachronous liver metastasis (MLM) in patients with gastric cancer.

In the CD68^+^ macrophage analysis, univariate regression analysis indicated that the area and perimeter of macrophages in the IF region, as well as the area and perimeter of macrophages in the tumor region, were potential factors. Multivariate regression analysis revealed that only the area and perimeter of macrophages in the IF region, the area of macrophages in the tumor region were independent predictive factors for MLM (**Table 3**). Then we developed the nomogram based on the above predictors (**Figure 3**), which enabled quantitative estimation of the risk of MLM in individual gastric cancer patients.

**Table 3:** Univariate and multivariate logistic regression in the training cohort (CD68^+^ macrophage group).

Characteristics	Non-MLM Group(n=100) (%)	MLM Group(n=63) (%)	Univariate analysis OR (95% CI)	P	Multivariate analysis OR (95% CI)	P
IF Area (μm^2^)			19.216(5.650-65.349)	<0.001	10.409(2.298-47.148)	0.002
<76.75	49 (49.0)	3 (4.8)				
≥76.75	51 (51.0)	60 (95.2)				
IF Perimeter (μm)			11.574(5.301-25.268)	<0.001	6.410(2.268-18.117)	<0.001
<35.77	71 (71.0)	11 (17.5)				
≥35.77	29 (29.0)	52 (82.5)				
Tumor Area (μm^2^)			0.581(0.306-1.102)	0.096	0.176(0.067-0.462)	<0.001
<71.35	36 (36.0)	31 (49.2)				
≥71.35	64 (64.0)	32 (50.8)				
Tumor Perimeter (μm)			0.567(0.297-1.080)	0.084		
<32.00	34 (34.0)	30 (47.6)				
≥32.00	66 (66.0)	33 (52.4)				
PS Area (μm^2^)			0.295(0.062-1.394)	0.123		
<74.13	90 (90.0)	61 (96.8)				
≥74.13	10 (10.0)	2 (3.2)				
PS Perimeter (μm)			1.572(0.729-3.389)	0.249		
<31.32	27 (27.0)	12 (19.0)				
≥31.32	73 (73.0)	51 (81.0)				

### Validation and assessment of the prediction model for MLM

**Figure 4:**
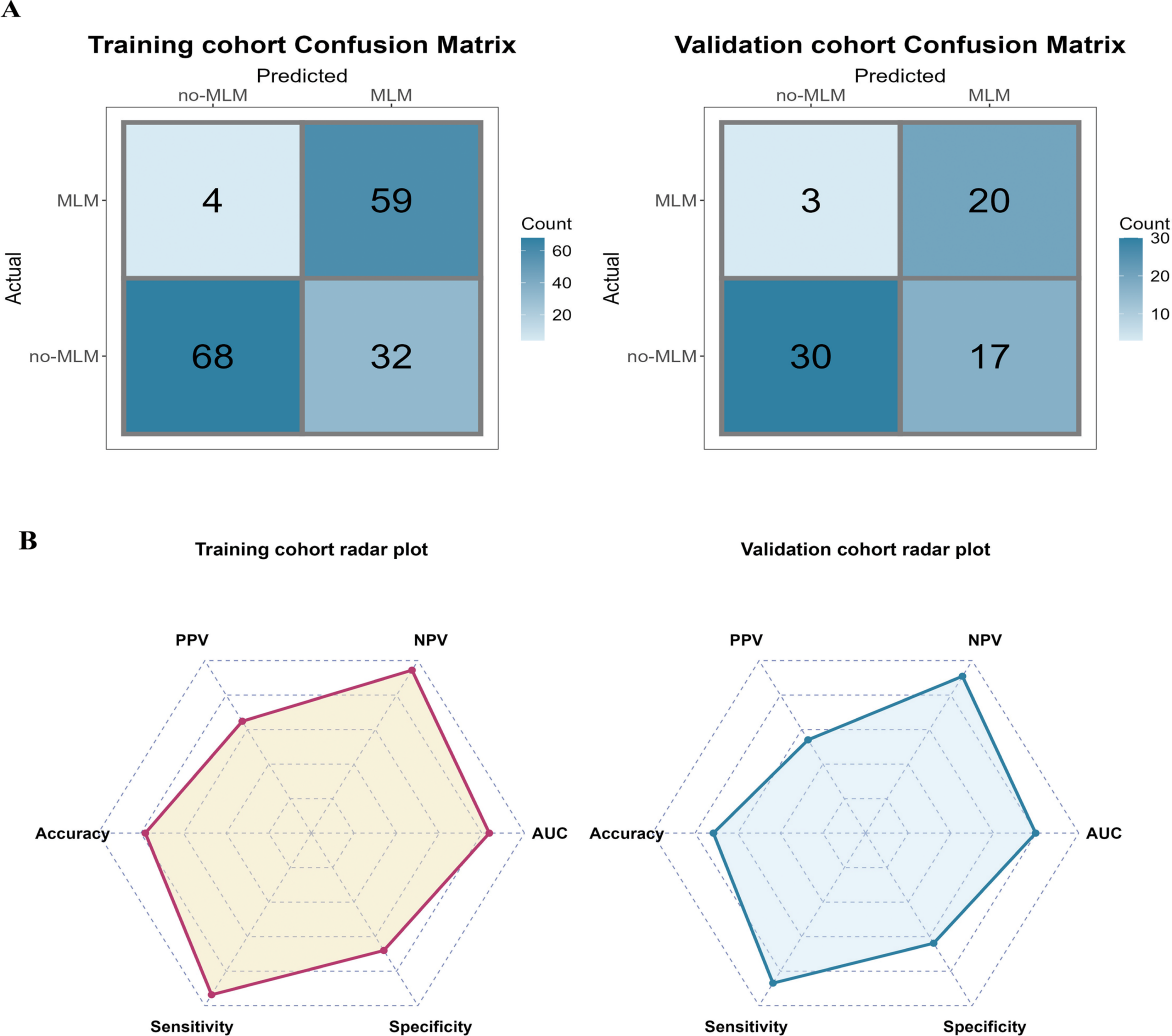
Classification performance of the nomogram in the training and validation cohorts. **(A)** Confusion matrices showing observed versus predicted metachronous liver metastasis (MLM) status. **(B)** Radar plots summarizing accuracy, sensitivity, specificity, positive predictive value (PPV), negative predictive value (NPV), and area under the curve (AUC).

To comprehensively evaluate the discriminative performance of the macrophage morphometry-based nomogram, confusion matrices were generated, and multiple quantitative classification metrics were calculated, including sensitivity, specificity, accuracy, positive predictive value (PPV), and negative predictive value (NPV), for both the training and validation cohorts (**Figure 4A**; **Table 4**). In the training cohort, 59 out of 63 patients with MLM and 68 out of 100 patients without MLM were correctly classified by the nomogram, with a corresponding sensitivity of 93.7%, specificity of 68.0%, accuracy of 77.9%, PPV of 64.8%, and NPV of 94.4%. In the validation cohort, 20 out of 23 MLM patients and 30 out of 47 non−MLM patients were correctly predicted, yielding a sensitivity of 87.0%, specificity of 63.8%, accuracy of 71.4%, PPV of 54.1%, and NPV of 90.9%. Radar plots were used to visualize the balanced overall performance of the nomogram across six key metrics (AUC, accuracy, sensitivity, specificity, PPV, NPV) in both cohorts, and consistent predictive performance with minimal overfitting was confirmed (**Figure 4B**).

**Table 4:** The predictive performance of the training cohort and the validation cohort.

Model	Accuracy	Sensitivity	Specificity	AUC (95%CI)	PPV	NPV
Training cohort	0.779	0.937	0.680	0.835(0.776-0.894)	0.648	0.944
Validation cohort	0.714	0.870	0.638	0.798(0.699-0.897)	0.541	0.909

**Figure 5:**
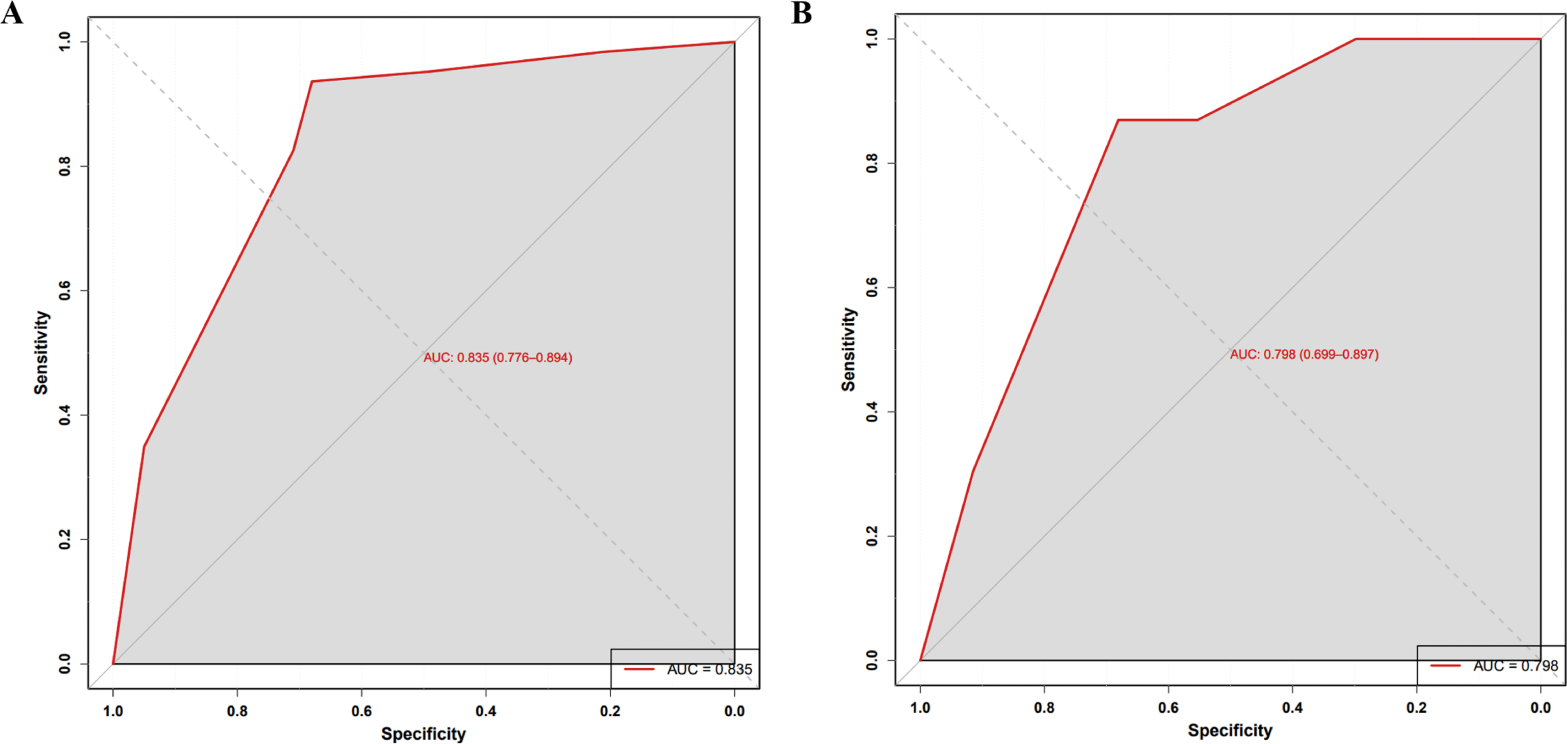
Receiver operating characteristic (ROC) curves of the predictive metachronous liver metastasis (MLM) model in the training cohort **(A)** and validation cohort **(B)**.

**Figure 6:**
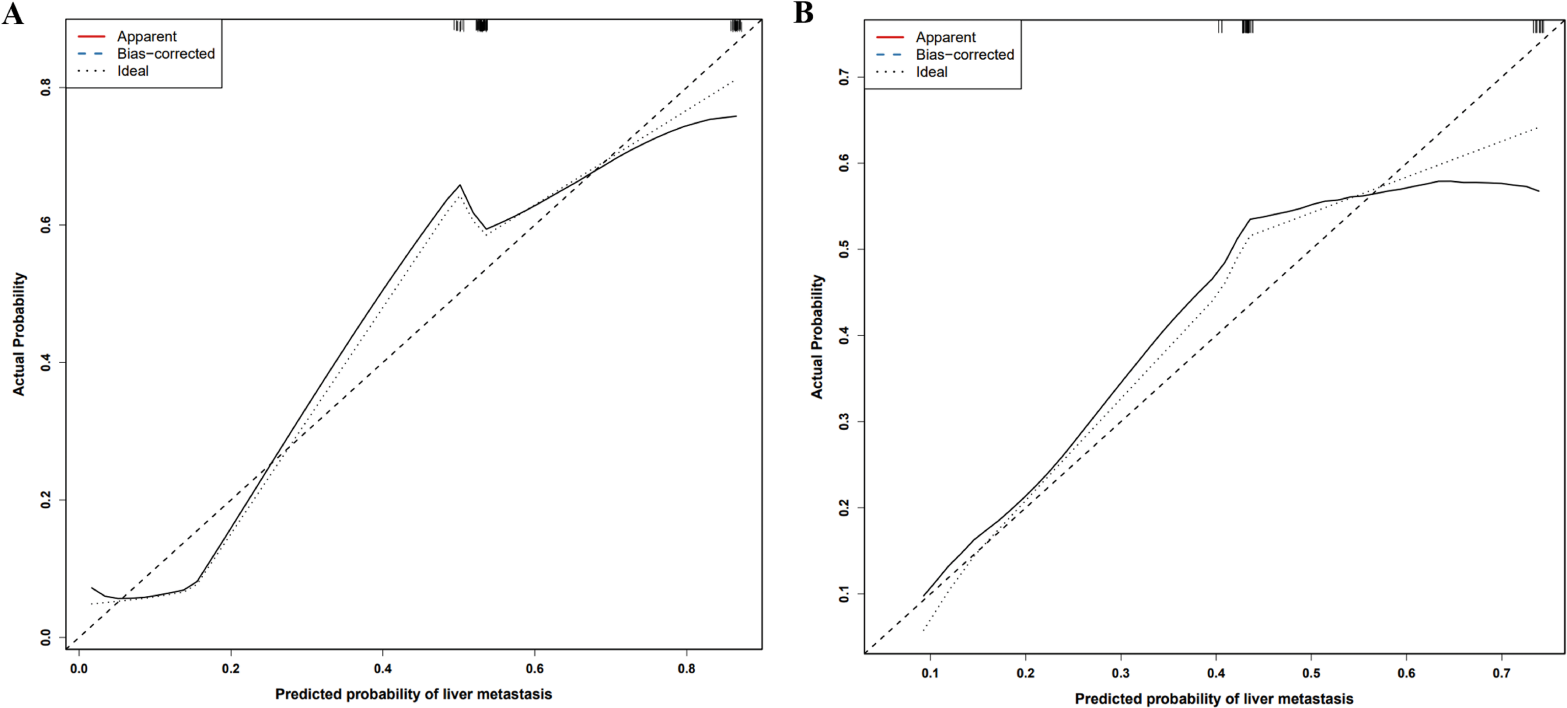
Calibration curves of the nomogram for predicting metachronous liver metastasis (MLM) **(A)** Training cohort. **(B)** Validation cohort.

**Figure 7:**
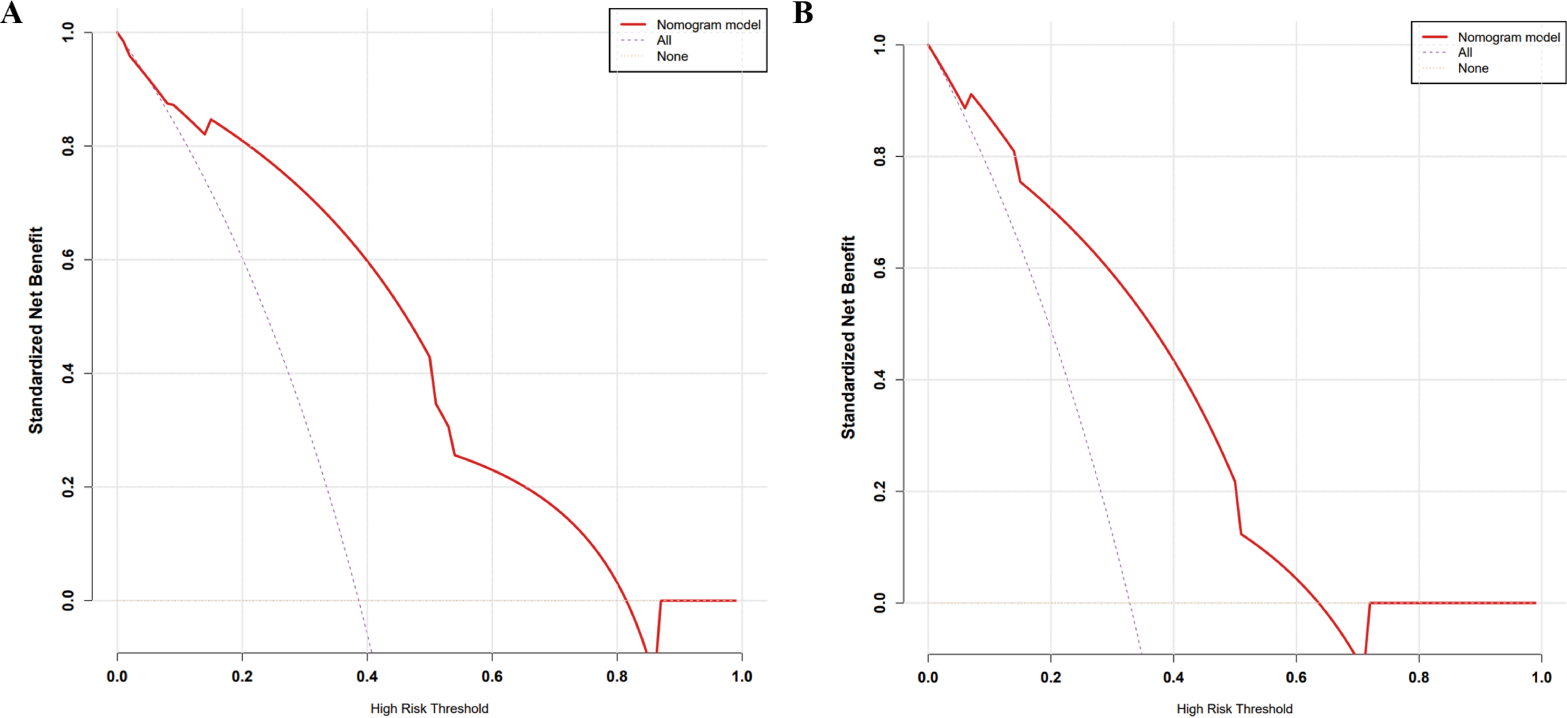
Decision curve analysis (DCA) of the nomogram for predicting metachronous liver metastasis (MLM) in the training cohort **(A)** and validation cohort **(B)**.

The model demonstrated predictive performance with AUC values of 0.835 (95%CI: 0.776-0.894) and 0.798 (95%CI: 0.699-0.897) in the training and validation cohorts, respectively (**Figure 5**). The calibration plots for the training cohort predicting MLM demonstrated a strong agreement between observed outcomes and model predictions (**Figure 6A**). Likewise, the calibration plots of the nomogram for predicting MLM in the validation cohort also indicated a high level of accuracy (**Figure 6B**). The DCA demonstrated that the nomogram exhibited superior clinical utility, confirming its robust clinical value in both the training and validation cohorts (**Figure 7**). In an exploratory supplementary analysis, joint evaluation of macrophage morphometric features also revealed a group-level difference between MLM and non-MLM cases in dimensionality reduction analyses, supporting the presence of broader macrophage-associated morphometric heterogeneity in the tumor microenvironment (**Supplementary Figure 7**).

## Discussion

MLM is a critical prognostic factor in gastric cancer, yet its relationship with the tumor immune microenvironment remains unexplored (8). Notably, to the best of our knowledge, this was the first study to leverage macrophage morphology within the tumor microenvironment for predicting MLM in gastric cancer. To investigate this, we characterized macrophages using CD68 and CD163 antibodies and found that morphological parameters of CD68^+^ macrophages served as independent predictors of MLM. Based on these findings, we established a morphology-based nomogram that showed favorable discrimination and calibration in internal validation. Notably, all included variables are readily obtainable, simple, and quantifiable in routine clinical practice.

CD68 is a classical pan-macrophage marker, while CD163 was included because previous studies indicated it is more evenly distributed on the cell surface, potentially making it suitable for morphometric features (16). In this retrospective study, CD163^+^ macrophage morphology did not yield multiple significant predictors, with only the area in the IF region showing association. This observation aligns with the understanding that macrophage functional plasticity, often reflected in their morphology, is influenced by the integrated signals of the broader tumor microenvironment rather than a single polarization pathway (27, 28). Therefore, CD68 was prioritized in the final nomogram based on model performance in this cohort rather than on the assumption that CD68 is universally superior for macrophage morphometric assessment. Importantly, our findings should not be interpreted as establishing CD68 as the optimal marker. Instead, under the current analytical framework, CD68-derived morphometric features demonstrated greater utility for multivariable prediction, whereas CD163-based parameters failed to provide comparable independent predictive value after adjustment.

As a key component of the tumor microenvironment (TME), macrophages have traditionally been classified into M1 and M2 subtypes based on their differentiation states and immunological functions (29). However, accumulating evidence suggests that this binary classification system is insufficient to account for the remarkable plasticity of macrophages and the resulting diversity of subtypes (30). Different macrophage subtypes exert distinct functional roles within the TME, which are correspondingly manifested in their morphological traits (18). Multicenter studies by Donadon et al. (16) and Costa et al. (19) demonstrated that macrophage morphometric features within the tumor microenvironment could serve as a simple readout of their diversity and allows to reliably stratify patient outcomes and predict disease recurrence after hepatectomy for colorectal liver metastases. However, their studies failed to consider the heterogeneity of macrophages across different histological compartments and only analyzed randomly selected macrophages from limited regions, raising concerns about sampling representativeness.

To address this gap, we adopted QuPath to perform a systematic and region-specific assessment of macrophage morphology. Inspired by Zhao et al (24), Gastric cancer tissues were grouped into seven classifications. Meanwhile, we trained an ANN model through learning, which were used to accurately segment different histological regions within primary gastric tumor specimens. We then used the software’s built-in positive cell detection module to extract morphometric parameters for all positive macrophages. For clinical interpretability, we focused on three key regions—the IF region, tumor region and PS region—and analyzed the area and perimeter of macrophages in these regions. This approach effectively mitigated randomness in cell selection and provided a more representative and reproducible assessment of macrophage morphology. Leveraging this method, we systematically examined the association between *in situ* morphometric features of macrophages and MLM risk, uncovering novel biomarkers with clinical applicability that surpasses conventional polarization markers. However, differences in specimen processing may affect the reproducibility and generalizability of morphology-based assessment. Variations in fixation, paraffin embedding, section thickness, immunohistochemical staining intensity, whole-slide scanning platforms, and region annotation practices may influence cell detection and morphometric measurements. Although we used a standardized institutional workflow and double-blinded review by experienced pathologists to improve consistency, the robustness of this image-analysis pipeline across external cohorts still requires further evaluation.

Previous studies have largely assessed the prognostic value of macrophages in gastric cancer based on their polarization (31), particularly the M2-polarized phenotype, along with associated the density (32), proportion (33), and expression levels (34). However, research on predicting MLM through macrophages remains limited, and studies employing readily obtainable macrophage morphological features are still lacking. Increased macrophage area or perimeter may reflect cytoskeletal remodeling (35), membrane spreading (36), adhesive interaction with the extracellular matrix, migratory behavior (37), and altered communication with neighboring tumor and stromal cells (38). These features may be particularly relevant at the invasive front, which represents a biologically active interface for tumor invasion and microenvironmental remodeling. The present study primarily focused on macrophage morphometric features rather than macrophage abundance. Therefore, the reported associations mainly reflect morphology-based parameters, although a potential interplay between macrophage density and morphology cannot be excluded. Our predictive model indicated that macrophage morphometric parameters contributed differently across distinct regions, reflecting regional heterogeneity. Specifically, larger macrophage area and perimeter in the IF region are associated with a higher risk of MLM, whereas smaller macrophage area in the tumor region corresponds to an increased MLM risk. Exploratory joint analysis of macrophage morphometric features further suggested that MLM is associated with broader shifts in macrophage-related morphometric patterns at the tumor microenvironment level. These patterns suggest that macrophages in different histological compartments may adopt distinct activation states or interact differently with local stromal and tumor signals.

This apparent discrepancy may be related to the context of their potential polarization states within distinct microenvironments. Naïve macrophages (M0) are typically small, round cells with minimal cytoplasm, whereas polarized macrophages often exhibit altered morphologies (39–41). M1-like macrophages are generally round and flattened, whereas M2-like macrophages exhibit an elongated morphology, often exhibiting more spread and complex morphology (42, 43). The IF region is associated with the recruitment of macrophages, which subsequently undergo M2-like polarization, further promoting local immunosuppression (25). Accordingly, the larger macrophage area and perimeter in the IF region may suggest a higher presence of macrophages with an activated or spread morphology, which is consistent with features described for M2-like polarization in the literature. This morphological profile could be indicative of a pro-metastatic microenvironment, contributing to a higher risk of MLM. Conversely, the tumor region may harbor a greater proportion of macrophages with anti-tumor properties, often classified as M1-like macrophages (27), which have been linked to improved recurrence-free survival (RFS) (44). A larger average cell area in the tumor region might indicate a greater presence of such activated, potentially anti-tumor macrophages, which could contribute to a protective effect against MLM. However, due to our sample condition and technical constraints, we did not perform immunofluorescence labeling to distinguish macrophage polarization states in the IF and tumor regions. Additional macrophage markers, such as CD206/MRC1 or HLA-DR, were not evaluated in the present study. Therefore, the interpretation that specific morphometric patterns may reflect M1-like or M2-like phenotypes should be regarded as a hypothesis-generating explanation rather than a definitive conclusion. Morphometric features may provide an indirect readout of macrophage functional heterogeneity, although the exact biological correlates require further mechanistic validation. Future studies incorporating multiplex phenotypic staining will be important to better link morphology with macrophage functional states.

Despite the promising findings of our study, several limitations should be acknowledged. First of all, it was a retrospective, single-center study with a limited sample size, and time-to-event analyses such as liver metastasis-free survival were not incorporated. The study could be affected by information biases and compromise model stability. Both the training and validation cohorts were derived from the same institution, the validation performed in this study should be regarded as internal validation. The absence of external validation may limit the generalizability of the model across different institutions, patient populations, tissue processing protocols, staining platforms, and digital pathology pipelines. Secondly, in the present study, PSM was mainly used to reduce substantial baseline imbalance and to allow a more focused exploration of the morphology-associated signal. This model did not integrate established clinicopathological predictors such as TNM stage. Accordingly, the current nomogram should be interpreted as an exploratory morphology-focused model rather than a definitive comprehensive clinical prediction tool. Thirdly, for clinical interpretability, continuous morphometric variables were dichotomized using cut-off values determined by the Youden index rather than as established biological thresholds. This approach could lead to information loss. We confined our analysis to three histological regions, namely the IF region, PS region and tumor region. Other regions were not analyzed and additional relevant information may be overlooked. Fourthly, this study was limited to macrophage morphology analysis and did not explore the mechanisms underlying macrophage morphometric changes in MLM.

In summary, our study for the first time demonstrates that macrophage morphology features in distinct histological regions of primary gastric cancer tissues, specifically the area and perimeter in the tumor invasive front and the area in the tumor region, are significantly associated with the risk of MLM. The morphology-based nomogram developed in this study may serve as an exploratory tool for postoperative risk stratification. Future validation through multicenter prospective studies using spatial transcriptomics and single-cell sequencing, and development of broadly applicable classifier models combining morphology with clinicopathological features are warranted to further refine and generalize these predictive insights in gastric cancer patients.

## Data Availability

The raw data supporting the conclusions of this article will be made available by the authors, without undue reservation.
