# Supplementary material for: Macrophage morphology in the tumor microenvironment predicts metachronous liver metastasis in gastric cancer: establishment and validation of a predictive model
Source: Front Immunol. 2026 Jun 5;17:1770436. doi: 10.3389/fimmu.2026.1770436 (PMC13279534; doi:10.3389/fimmu.2026.1770436)
Supplement: Supplementary file 1 [file SupplementaryFile1.pdf]

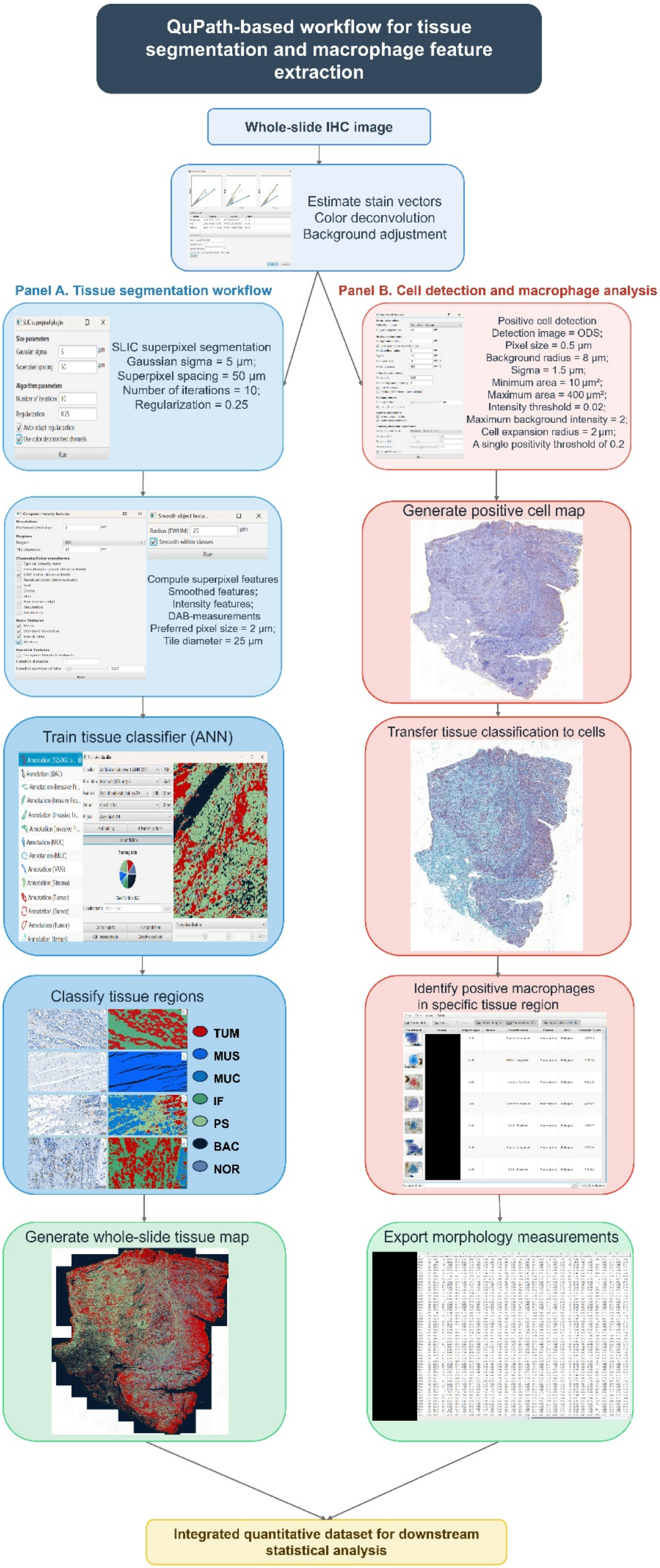
QuPath-based workflow for tissue segmentation and macrophage feature extraction
Whole-slide IHC image
Estimate stain vectors
Color deconvolution
Background adjustment
Panel A. Tissue segmentation workflow
Panel B. Cell detection and macrophage analysis
SLIC superpixel segmentation
Gaussian sigma = 5 µm;
Superpixel spacing = 50 µm
Number of iterations = 10;
Regularization = 0.25
Positive cell detection
Detection image = ODS;
Pixel size = 0.5 µm
Background radius = 8 µm;
Sigma = 1.5 µm;
Minimum area = 10 µm²;
Maximum area = 400 µm²;
Intensity threshold = 0.02;
Maximum background intensity = 2;
Cell expansion radius = 2 µm;
A single positivity threshold of 0.2
Compute superpixel features
Smoothed features;
Intensity features;
DAB-measurements
Preferred pixel size = 2 µm;
Tile diameter = 25 µm
Generate positive cell map
Train tissue classifier (ANN)
Transfer tissue classification to cells
Classify tissue regions
TUM
MUS
MUC
IF
PS
BAC
NOR
Identify positive macrophages
in specific tissue region
Generate whole-slide tissue map
Export morphology measurements
Integrated quantitative dataset for downstream statistical analysis

Supplementary Figure 1: QuPath-based workflow for tissue segmentation and macrophage feature extraction from whole-slide immunohistochemistry (IHC) images.

The analytical pipeline consisted of two parallel modules initiated from the same whole-slide immunohistochemistry (IHC) image after stain vector estimation by color deconvolution and background adjustment. Panel A illustrates the tissue segmentation workflow. First, SLIC superpixel segmentation was performed using a Gaussian sigma of 5 μm, superpixel spacing of 50 μm, 10 iterations, and a regularization parameter of 0.25. Superpixel-level measurements were then computed, including smoothed features, intensity features, and DAB-based measurements, with a preferred pixel size of 2 μm and a tile diameter of 25 μm. Based on these features, an artificial neural network (ANN)-based tissue classifier with default multiscale features at moderate resolution (1.94 μm/px) was trained to categorize tissue regions into tumor (TUM), smooth muscle (MUS), mucin (MUC), invasive front (IF), peritumoral stroma (PS), background (BAC), and normal mucosa (NOR), generating a whole-slide tissue map. Panel B shows the cell detection and macrophage analysis workflow. Positive cell detection was performed using the optical density sum image with a requested pixel size of 0.5 μm, background radius of 8 μm, sigma of 1.5 μm, minimum and maximum areas of 10 and 400 μm^2, respectively, intensity threshold of 0.02, maximum background intensity of 2, cell expansion radius of 2 μm, and a single positivity threshold of 0.2. A positive cell map was generated, after which tissue classifications were transferred to detected cells, enabling identification of positive macrophages within specific tissue compartments. Cell-level morphology and other quantitative measurements were then exported and integrated with the tissue map for downstream statistical analysis.

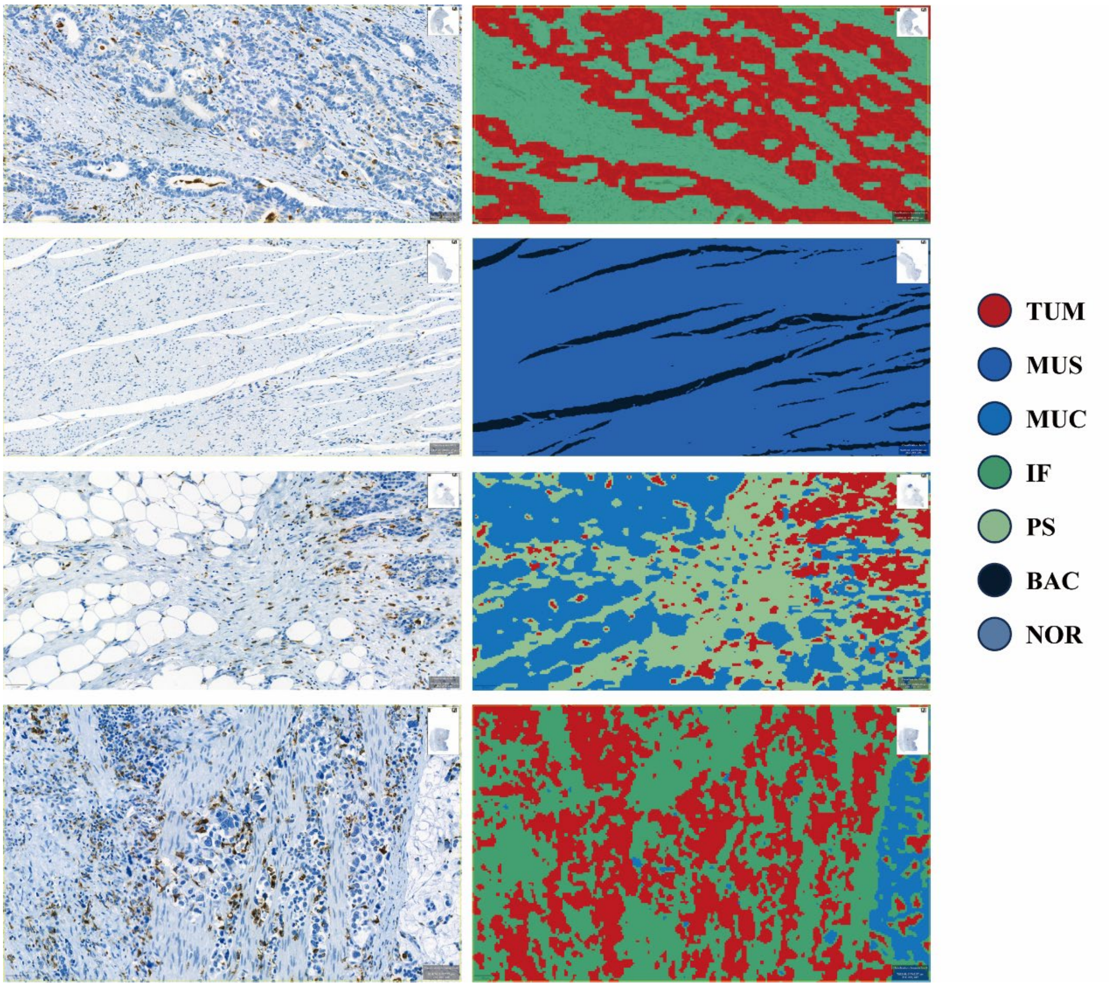

Supplementary Figure 2. Tissue region annotation and artificial neural network classifier training workflow in QuPath.

Representative immunohistochemistry (IHC) images of gastric cancer tissues and corresponding manual annotations used for ANN classifier training are shown. Annotated histological regions included tumor (TUM), invasive front (IF), peritumoral stroma (PS), smooth muscle (MUS), mucin (MUC), normal mucosa (NOR), and background (BAC).

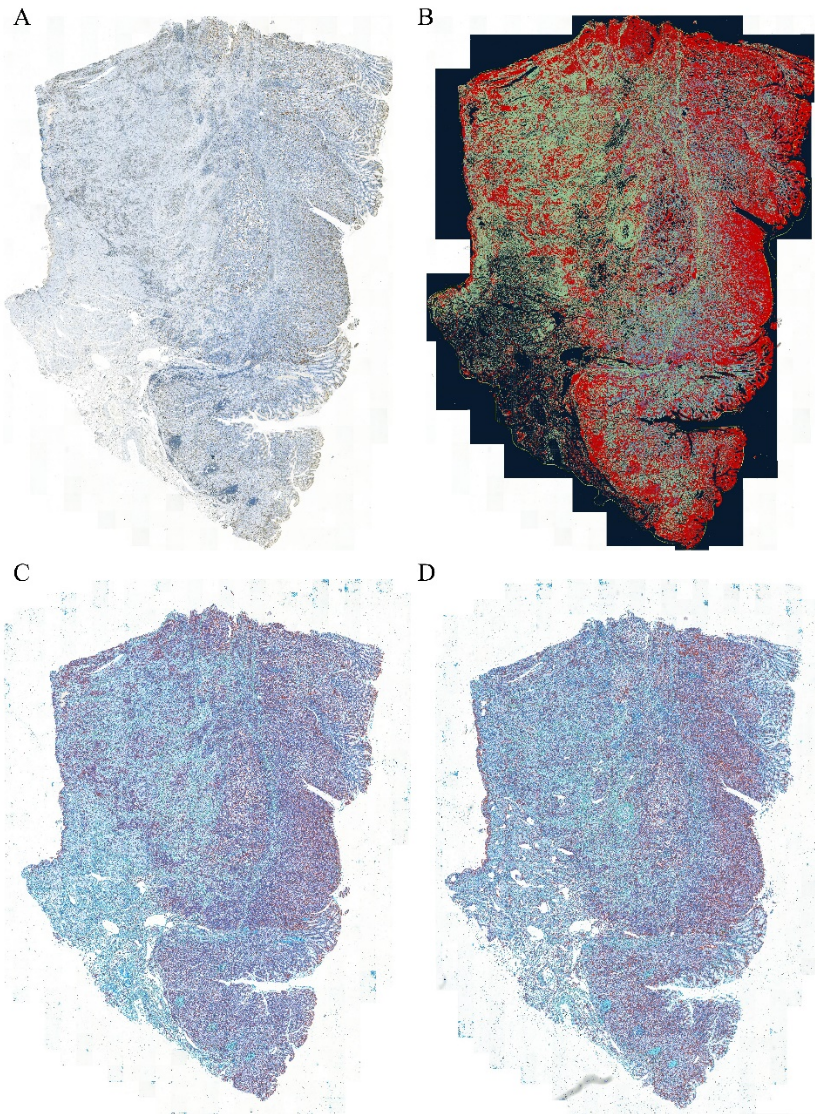

Supplementary Figure 3. Representative whole-slide immunohistochemical images and QuPath-based macrophage segmentation workflow for CD68 and CD163 staining.

(A) Original whole-slide immunohistochemical image of primary gastric cancer tissue.(B) Corresponding tissue-compartment segmentation map generated by the artificial neural network (ANN) classifier. (C) Whole-slide view of detected $CD68^{+}$ macrophages, mapped to individual histological compartments. (D) Whole-slide view of detected $CD163^{+}$ macrophages with identical tissue-region stratification.

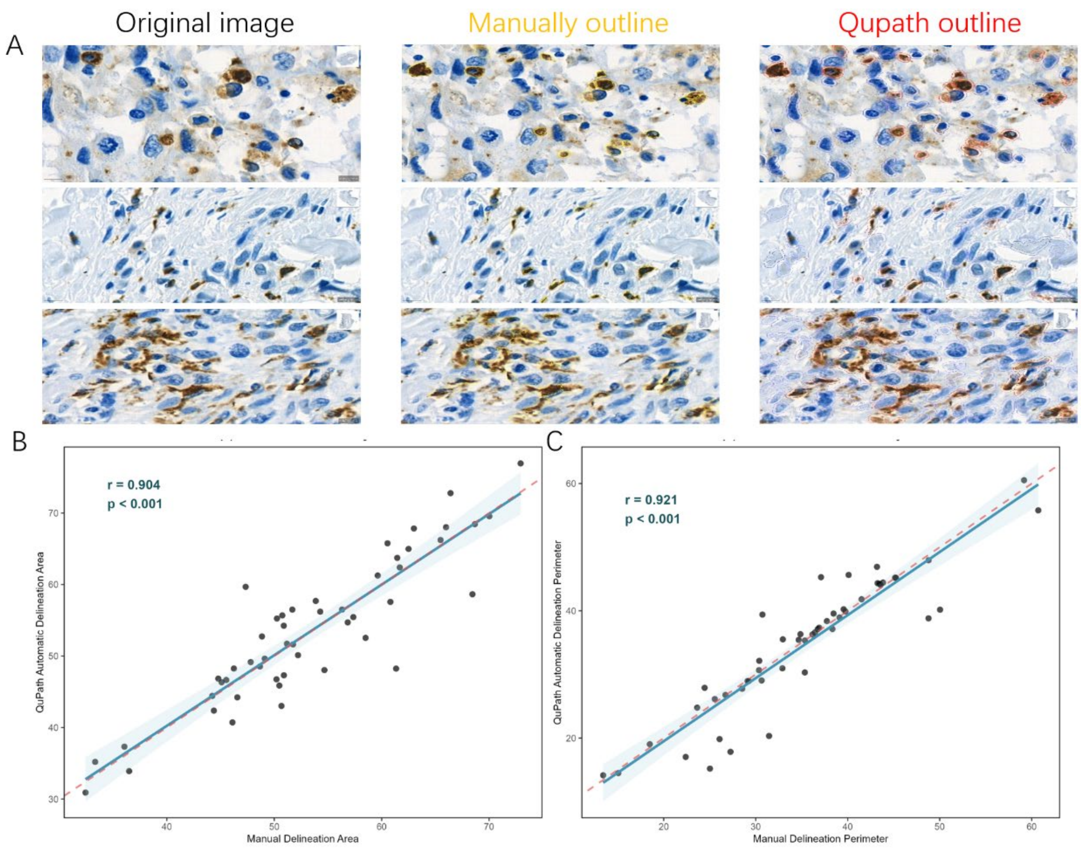

Supplementary Figure 4: Correlation analysis of macrophage area and perimeter measured by manual delineation and QuPath-based automated segmentation.

(A) Representative immunohistochemical image showing a macrophage analyzed by both manual delineation (orange outline) and QuPath automated segmentation (red outline). Scale bar, 10 μm. (B, C) Scatter plots demonstrating a strong positive correlation between the automated and manual methods for measuring macrophage area (B; $r = 0.904$, $p < 0.001$) and perimeter (C; $r = 0.921$, $p < 0.001$). Pearson's correlation coefficient was used.

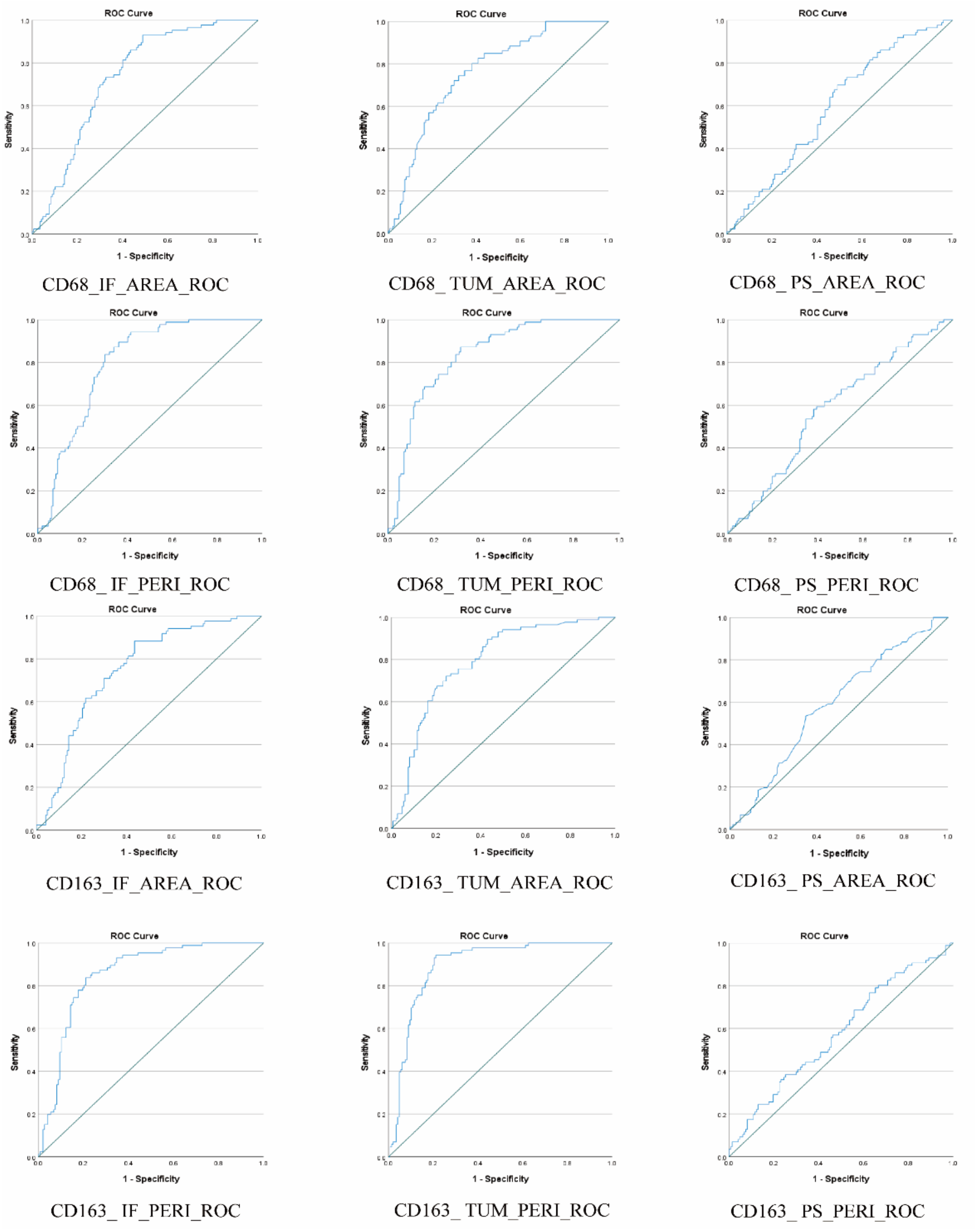

Supplementary Figure 5. Receiver operating characteristic (ROC) curves of CD68- and CD163-based macrophage morphometric parameters for predicting metachronous liver metastasis (MLM).

ROC curves were generated in the training cohort for macrophage area and perimeter features of $CD68^+$ and $CD163^+$ macrophages in three histological compartments: invasive front (IF), tumor region (TUM), and peritumoral stroma (PS). Each curve represents the diagnostic performance of an individual morphometric parameter for identifying patients at risk of MLM after gastric cancer resection.

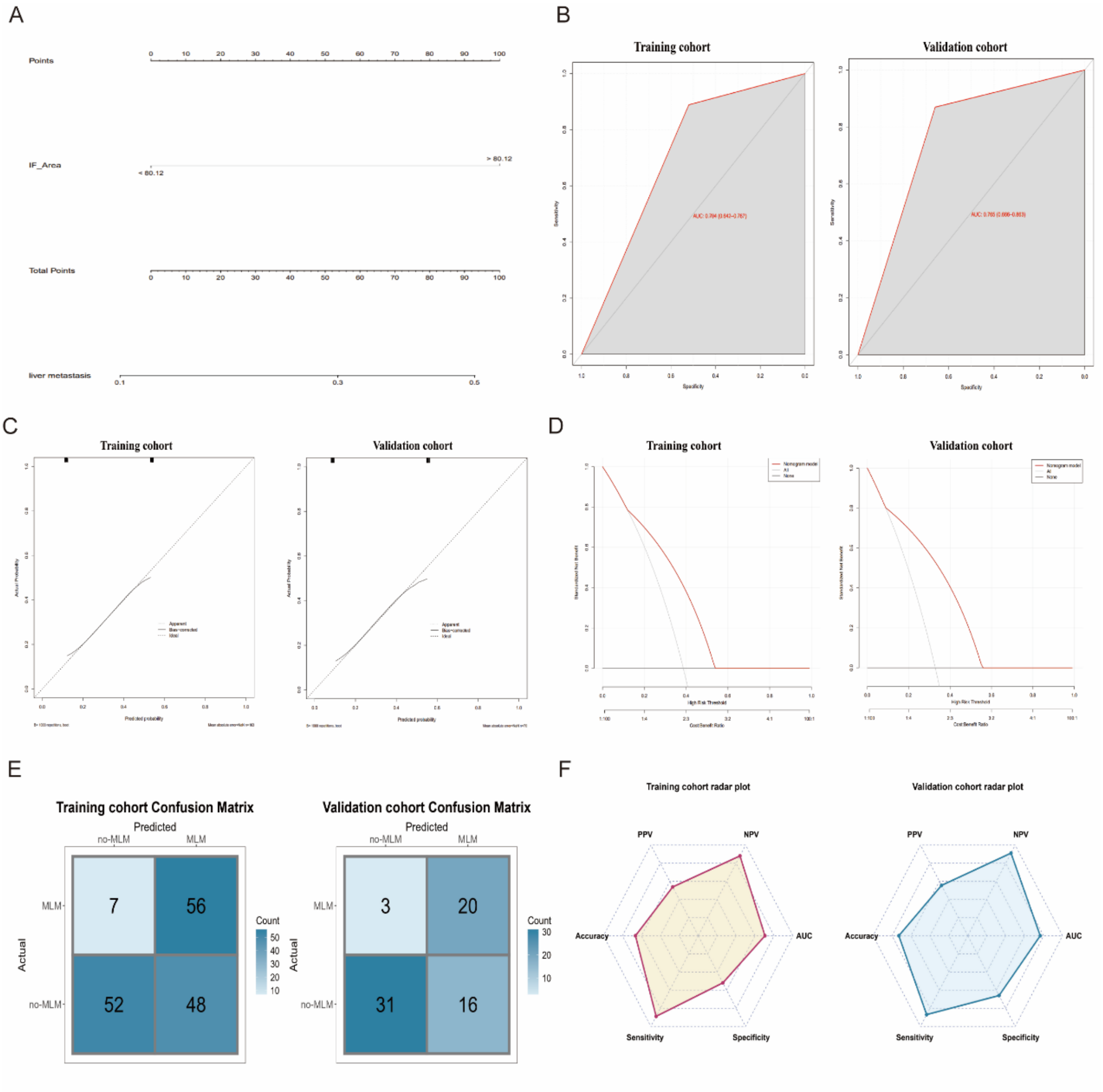

Supplementary Figure 6: Exploratory development and validation of a CD163-based model for predicting metachronous liver metastasis (MLM) in gastric cancer.

(A) Exploratory model/nomogram for predicting metachronous liver metastasis in patients with gastric cancer. (B) Receiver operating characteristic (ROC) curves in the training and validation cohorts. (C) Calibration curves comparing predicted versus observed probabilities in both cohorts. (D) Decision curve analysis (DCA) of the nomogram model for predicting MLM in the training and validation cohort. (E) Confusion matrices showing actual versus predicted MLM status in the training and validation cohorts. (F) Radar plots summarizing accuracy, sensitivity, specificity, positive predictive value (PPV), negative predictive value (NPV), and area under the curve (AUC) in the training and validation cohorts. This analysis is presented as a supplementary exploratory comparison and does not represent the final model reported in the main text.

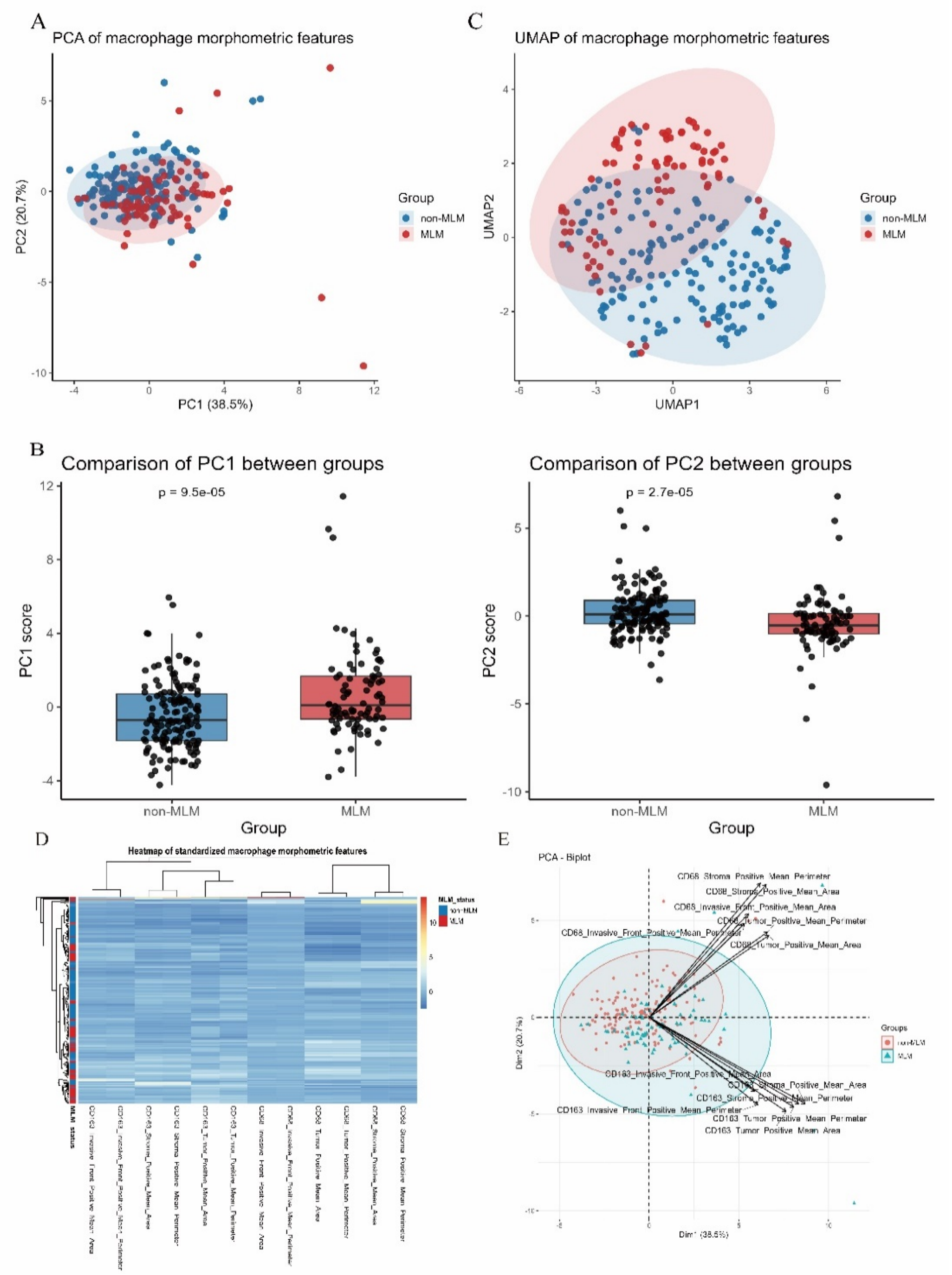

Supplementary Figure 7: Exploratory joint analysis of macrophage morphometric features in relation to metachronous liver metastasis (MLM).

(A) Principal component analysis (PCA) score plot based on combined macrophage morphometric features. (B) Comparison of principal component scores (PC1 and PC2) between the non-MLM and MLM groups. (C) Uniform manifold approximation and projection (UMAP) of the combined macrophage morphometric features, showing the overall sample distribution in a nonlinear low-dimensional space. (D) Heatmap of standardized macrophage morphometric features across individual samples. (E) PCA biplot showing the contribution of representative morphometric variables to the principal component structure.

Supplementary Table 1. Baseline clinical characteristics before propensity score matching (PSM)

Characteristics	Non-MLM(n=1114) (%)	MLM(n=114) (%)	P	SMD
Age (year (SD))	58.31±10.37	59.47±10.39	0.254	0.112
Tumor size (cm (SD))	3.70±2.10	5.56±2.45	<0.001	0.817
Gender			0.017	0.268
Male	853 (76.6)	99 (86.8)		
Female	261 (23.4)	15 (13.2)		
Tumor location			<0.001	0.364
Upper third	312 (28.0)	36 (31.6)		
Middle third	269 (24.1)	24 (21.1)		
Lower third	522 (46.9)	45 (39.5)		
Entire	11 (1.0)	9 (7.9)		
Pathological type			0.006	0.407
Well differentiated	38 (3.4)	0 (0.0)		
Moderately differentiated	299 (26.8)	18 (15.8)		
Poorly differentiated	638 (57.3)	82 (71.9)		
Signet ring cell/Mucinous /undifferentiated	139 (12.5)	14 (12.3)		
T stage			<0.001	1.107
T1	342 (30.7)	2 (1.8)		
T2	178 (16.0)	8 (7.0)		
T3	351 (31.5)	36 (31.6)		
T4	243 (21.8)	68 (59.6)		

N stage			<0.001	1.163
N0	582 (52.2)	16 (14.0)		
N1	222 (19.9)	14 (12.3)		
N2	184 (16.5)	25 (21.9)		
N3	126 (11.3)	59 (51.8)		
TNM stage			<0.001	1.247
I	428 (38.4)	3 (2.6)		
II	355 (31.9)	22 (19.3)		
III	331 (29.7)	89 (78.1)		
Neural invasion			<0.001	0.551
Negative	331 (29.7)	10 (8.8)		
Positive	783 (70.3)	104 (91.2)		
Lymphovascular invasion			<0.001	0.758
Negative	558 (50.1)	19 (16.7)		
Positive	556 (49.9)	95 (83.3)		
Surgical method			<0.001	0.450
Proximal Gastrectomy	132 (11.8)	7 (6.1)		
Distal Gastrectomy	596 (53.5)	43 (37.7)		
Total Gastrectomy	386 (34.6)	64 (56.1)		

Supplementary Table 2. Diagnostic performance metrics of macrophage morphometric parameters derived from ROC analysis in the training cohort.

Characteristics	cut-off value	sensitivity	specificity	Youden index	AUC
CD68_IF_AREA	76.75μm^2	0.930	0.510	0.440	0.737
CD68_IF_PERI	35.77μm	0.837	0.701	0.538	0.800
CD68_TUM_AREA	71.35μm^2	0.744	0.680	0.424	0.760
CD68_TUM_PERI	32.00μm	0.872	0.687	0.559	0.837
CD68_PS_AREA	74.13μm^2	0.698	0.510	0.208	0.595
CD68_PS_PERI	31.32μm	0.581	0.619	0.200	0.586
CD163_IF_AREA	80.12μm^2	0.884	0.565	0.448	0.750
CD163_IF_PERI	36.96μm	0.837	0.789	0.626	0.853
CD163_TUM_AREA	77.92μm^2	0.721	0.755	0.476	0.790
CD163_TUM_PERI	32.20μm	0.942	0.789	0.731	0.893
CD163_PS_AREA	70.50μm^2	0.535	0.653	0.188	0.588
CD163_PS_PERI	33.47μm	0.767	0.374	0.142	0.578

Supplementary Table 3. Univariate and multivariate logistic regression in the training cohort (CD163^{+} macrophage group)

Characteristics	Non-MLM Group(n=100) (%)	MLM Group(n=63) (%)	Univariate analysis OR (95% CI)	P	Multivariate analysis OR (95% CI)	P
IF Area (μm^2)			8.667(3.601-20.857)	<0.001	11.066(3.663-33.429)	<0.001
< 80.12	52 (52.0)	7 (11.1)				
≥80.12	48 (48.0)	56 (88.9)				
IF Perimeter (μm)			18.000(7.784-41.626)	<0.001		
< 36.96	75 (75.0)	9 (14.3)				
≥36.96	25 (25.0)	54 (85.7)				
Tumor Area (μm^2)			48.545(18.895-124.722)	<0.001		
< 77.92	89 (89.0)	9 (14.3)				
≥77.92	11 (11.0)	54 (85.7)				
Tumor Perimeter (μm)			2.317(1.148-4.679)	0.019		
< 32.20	42 (42.0)	15 (23.8)				
≥32.20	58 (58.0)	48 (76.2)				
PS Area (μm^2)			1.393(0.730-2.658)	0.315		
< 70.50	65 (65.0)	36 (57.1)				
≥70.50	35 (35.0)	27 (42.9)				
PS Perimeter (μm)			1.624(0.849-3.107)	0.143		
< 33.47	67 (67.0)	35 (55.6)				

≥33.47	33 (33.0)	28 (44.4)
